# The expression and potential function of bone morphogenetic proteins 2 and 4 in bovine trophectoderm

**DOI:** 10.1186/1477-7827-10-12

**Published:** 2012-02-13

**Authors:** Kathleen A Pennington, Alan D Ealy

**Affiliations:** 1 Department of Animal Sciences, University of Florida, PO Box 110910, Gainesville, FL, USA

**Keywords:** Paracrine factor, Placenta, Conceptus, Interferon-tau

## Abstract

**Background:**

Bone morphogenetic proteins (BMPs) were first described for their roles in bone formation, but they now also are known to possess additional activities, including those relating to embryogenesis. The objectives of this work were to 1) determine if peri-attachment bovine conceptuses and bovine trophoblast cells (CT1) contain transcripts for *BMP2 *and *4*, an innate inhibitor noggin (*NOG*), and BMP2/4 receptors (*BMPRII*, *ACVR1*, *BMPR1A*, *BMPR1B*), and 2) determine if BMP2 or 4 supplementation to CT1 cells affects cell proliferation, differentiation or trophoblast-specific gene expression.

**Methods:**

RNA was isolated from day 17 bovine conceptuses and CT1 cells. After RT-PCR, amplified products were cloned and sequenced. In other studies CT1 cells were treated with BMP2 or 4 at various concentrations and effects on cell viability, cell differentiation and abundance of IFNT and CSH1 mRNA were evaluated.

**Results:**

Transcripts for *BMP2 *and *4 *were detected in bovine conceptuses and CT1 cells. Also, transcripts for each BMP receptor were detected in conceptuses and CT1 cells. Transcripts for *NOG *were detected in conceptuses but not CT1 cells. Cell proliferation was reduced by BMP4 but not BMP2 supplementation. Both factors reduced *IFNT *mRNA abundance but had no effect on *CSH1 *mRNA abundance in CT1 cells.

**Conclusions:**

The BMP2/4 ligand and receptor system presides within bovine trophectoderm prior to uterine attachment. BMP4 negatively impacts CT1 cell growth and both BMPs affect IFNT mRNA abundance.

## Background

Bone morphogenetic proteins (BMPs) are part of the transforming growth factor-β (TGFβ) superfamily of paracrine factors [1,2]. The BMPs mediate various physiological and developmental processes, including placental development [3]. The BMP4 family of factors, which include BMPs 2 and 4, appear to be especially important in placental development. In the mouse, conceptuses lacking BMP4 undergo developmental arrest at days 6.5-9.5 and lack mesoderm and placental vasculature [4-6]. Mesoderm formation also is absent in mice lacking Bmpr2, the Type II receptor for BMP4 [7]. Interestingly, Bmpr2 null mice have a more severe phenotype than mice lacking BMP4, suggesting the partial rescue of the BMP4 null phenotype by other BMPs, such as BMP2 [8].

Bone morphogenetic proteins 2 and 4 also regulate trophoblast lineage development and differentiation. Trophoblast development from human embryonic stem cells is induced by BMP2 and 4 [9,10]. In cattle, BMP4 supplementation improves the formation of trophoblast cell outgrowths from blastocysts [11]. Moreover, trophoblast cell lines generated from these outgrowths produce a multitude of factors, including interferon-tau (IFNT), the maternal recognition of pregnancy factor in ruminants that is secreted from mononucleated cells (MNCs) before placental attachment to the uterine lining [12]. Some of the cell lines derived by BMP4 treatment contain large quantities of *IFNT *mRNA whereas other lines contain little *IFNT *and instead contain greater quantities of transcripts detected in differentiated, binucleate cells (BNCs) after placental attachment [11]. It remains unclear if BMP4 may promote trophoblast cell differentiation during culture.

The overall goal of this work was to describe the expression and potential actions of the BMP4 ligand-receptor system during the pre- and peri-attachment period of bovine conceptus development. In the first set of studies, transcript patterns were determined for *BMP2*, *BMP4*, noggin (*NOG*; a BMP2/4 inhibitor) and BMP2/4 receptors (*BMPRII, ACVR1, BMPR1A, BMPR1B*) in peri-attachment bovine conceptuses (day 17 of gestation) and CT1 cells, a bovine trophoblast cell line that produces IFNT but not BNC marker genes [13,14]. In the second set of studies, CT1 cells were treated with BMP2 or 4 to explore whether these factors impact growth, differentiation and gene expression of these cells.

## Methods

### Animal use and tissue collection

All animal use was completed with the approval of the Institutional Animal Care and Use Committee at the University of Florida. Healthy, non-lactating Holstein cows (n = 12) were housed at the University of Florida Dairy Unit (Hague, FL) and fed a diet to meet their maintenance requirements (mixed ration containing corn, soybean meal and haylage along with continuous pasture grazing). Elongated conceptuses were collected at day 17 post-estrus as described previously [15]. In brief, cows were superovulated, bred via artificial insemination and slaughtered by captive bolt trauma and exsanguination at the University of Florida Meats Laboratory at day 17 post-estrus. Reproductive tracts were excised and uterine horns were flushed with Dulbecco's phosphate-buffered saline (DPBS; Life Technologies, Grand Island, NY) to collect conceptuses.

Endometrial samples were collected from non-superovulated cows that were bred and verified pregnant by the presence of a conceptus as described previously [16], and RNA was extracted using the Trizol reagent and PureLink Purification Columns (Life Technologies).

### End-point RT-PCR

RNA concentration and integrity were evaluated using a NanoDrop 2000 Spectrophotometer (Thermo Scientific). Samples (250 ng/reaction; A_260_/_280 _ratio ≥ 1.8) were incubated in RNase-free DNase (New England Biolabs, Ipswich, MA) for 30 min at 37°C followed by heat inactivation for 10 min at 75°C. The Superscript III First Strand Synthesis System (Life Technologies) and random hexamers were used for reverse transcription at 50°C for 60 min. ThermalAce DNA Polymerase (Life Technologies) was used to amplify DNA (35 cycles; 95°C - 1 min, 55 to 59°C for 1 min [depending on the primer pair; Table 1], 74°C - 1 min). Non-reverse transcribed DNase-treated RNA was included as a control for genomic DNA contamination. Amplified products were electrophoresed on a 1% [w/v] agarose gel containing 0.5 μg/ml ethidium bromide and visualized by UV light. Buffer and residual primers were removed from PCR Products by using the PureLink PCR Purification Kit (Life Technologies) and DNA sequencing was completed at the University of Florida DNA Core Facility.

**Table 1:** Primers used for end-point RT-PCR

Gene of Interest	Primer	Sequence (5'-3')
*BMP2*	Forward Reverse	CTTAGACGGTCTGCGGTCTC CGAAGCTCTCCCACCTACTG

*BMP4*	Forward Reverse	TGAGCCTTTCCAGCAAGTTT TACGATGAAAGCCCTGATCC

*NOG*	Forward Reverse	GAACACCCGGACCCTATCTT ATGGGGTACTGGATGGGAAT

*BMPRII*	Forward Reverse	AGACTGTTGGGACCAGGATG GTCTGGCCCACTGAATTGTT

*ACVR1*	Forward Reverse	AAATGGGATCGCTGTACGAC CTGTGAGTCTGGCAGATGGA

*BMPR1A*	Forward Reverse	CAGGTTCCTGGACTCAGCTC CACACCACCTCACGCATATC

*BMPR1B*	Forward Reverse	AGGTCGCTATGGGGAAGTTT CTCCCAAAGGATGAGTCCAA

*ACTB^a^*	Forward Reverse	CTGTCCCTGTATGCCTCTGG AGGAAGGAAGGCTGGAAGAG

^a^[15]

### CT1 culture

The bovine trophectoderm cell line, CT1, previously developed and characterized by Talbot et. al [17] was cultured as described previously [15-17]. In brief, cells were propagated in Dulbecco's Modified Eagle's Medium (DMEM) with high glucose (5.5 mM) containing 10% fetal bovine serum and other supplements (100 μM non-essential amino acids, 2 mM glutamine, 2 mM sodium pyruvate, 55 μM β-mercaptoethanol, 100 U/ml penicillin G, 100 μg/ml streptomycin sulfate, and 250 ng/ml amphoterin B; each from Life Technologies) on Matrigel™ Basement Membrane Matrix (appr. 1 mg/ml; BD Biosciences, Bedford, MA) at 38.5°C in 5% CO_2 _in air. Cells were passaged manually by scraping cells from the plates and passing them through a small-bore needle to produce small clumps of cells. On the day before supplementing BMPs, medium was replaced with medium lacking FBS (serum-free medium) and containing a serum substitute (ITS; Life Technologies). All other supplements were kept constant. Serum-free medium was exchanged the next day immediately before adding BMP treatments. Recombinant human (rh) BMP2 or rhBMP4 (R&D Systems, Minneapolis, MN) was reconstituted according to manufacturer's instructions and stored at -20°C in single-use aliquots. All treatments were prepared in 0.1% [w/v] BSA solution in DMEM on the day of supplementation. The controls contained vehicle only.

### CT1 cell number assay

CT1 cells were seeded at low confluency (1 × 10^5 ^cells/ml) into 24-well Matrigel™ coated plates (1 mg/ml) and incubated for 48 h to permit cell adherence to the Matrigel. Serum-free medium containing 0, 0.1, 1, 10, or 100 ng/ml rhBMP2 or rhBMP4 was added to cultures (4 wells/treatment; 3 replicate studies). Forty-eight hours later the reduction of a tetrazolium compound (MTS) into a colored formazon product was used to quantify viable cell numbers. Color intensity at 490 nm was measured after 30 min incubation (Cell Titer 96 Aqueous One Solution Cell Proliferation Assay; Promega Corp., Madison, WI). Visual assessment at the time of the MTS treatment indicated that confluency was > 50% in each replicate study.

### Quantitative (q) RT-PCR

RNA concentration and integrity were evaluated, and samples (10 ng/reaction; A_260_/_280 _ratio ≥ 1.8) were incubated in RNase-free DNase (New England Biolabs, Ipswich, MA) for 30 min at 37°C followed by heat inactivation for 10 min at 75°C. Reverse transcription and TaqMan PCR was completed as described previously [18] using an *IFNT *primer-probe set that recognizes all known bovine *IFNT *isoforms (a MNC-specific transcript) or a primer-probe set specific for *CSH1 *(chorionic somatomammotropic hormone; also known as placental lactogen), a binucleate cell-specific transcript [19] (Table 2). Both probes were labeled with a fluorescent 5' 6-FAM reporter dye and 3' TAMRA quencher (Life Technologies). Reactions also examined *18S *RNA abundance (reference control) (*18S *RNA Control Reagent Kit, 5'-VIC-labeled probe, Life Technologies). This reference was chosen based on previous studies [18] and because its mRNA concentrations were not impacted by treatments. After an initial activation/denaturation step (50°C for 2 min; 95°C for 10 min), 40 cycles of a two-step amplification procedure (60°C for 1 min; 95°C for 15 s) was completed using a 7300 Real-Time PCR System (Applied Biosystems, Life Technologies). Each RNA sample was analyzed in triplicate. A DNase-treated RNA not exposed to reverse transcriptase was included for each sample ensures samples were free of genomic DNA contamination. The ΔC_t _method was used to examine the relative abundance of *IFNT *and *CSH1 *transcripts with that of *18S *RNA [18].

**Table 2:** Primer and probe sets used for qRT-PCR

Gene of Interest	Primer/ Probe	**Sequence (5'-3')**^**a**^
*IFNT^b^*	Forward Reverse Probe	TGCAGGACAGAAAAGACTTTGGT CCTGATCCTTCTGGAGCTGG TTCCTCAGGAGATGGTGGTAGGGCA

*CSH1*	Forward Reverse Probe	GTGGATTTGTGACCTTGTTCGA CCTGGCACAAGAGTAGATTTGACA TCCTGCCTGCTCCTGCTGCTGGTA

^a^Each probe was synthesized with a 6-FAM reporter dye and TAMRA quencher

^b^[15]

### Statistical analyses

All analyses were completed by using the General Linear Model of analysis of variance from the Statistical Analysis System (SAS Institute, Cary, NC). For qRT-PCR analysis, ΔC_T _values were used for analyses and data were graphed by examining fold-effect [15,16]. Results are presented as arithmetic means ± SEM.

## Results and discussion

The first set of studies examined whether the various components of the BMP2/4 ligand-receptor system were expressed in elongated conceptuses and CT1 cells. A single sample of RNA collected from endometria of pregnant cows was included as a positive control.

**Figure 1:**
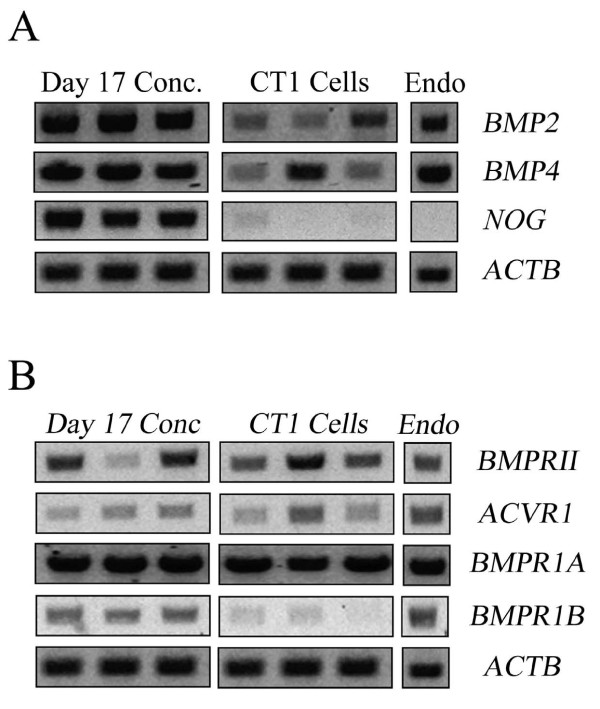
**End-point RT-PCR analysis of transcripts for bovine BMP ligands and receptors in the peri-attachment conceptus (day 17 post-insemination), trophoblast cells (CT1) and endometrium (Endo.)**. After RT-PCR, reactions were electrophoresed in 1% [w/v] agarose containing ethidium bromide and amplicons were visualized using UV light. Three different conceptus and CT1 RNA preparations were used in each reaction but only a single endometrium RNA sample collected from cyclic cows at day 17 post-estrus was included. Panel A depicts BMP ligands and inhibitors. Panel B depicts BMP2/4 receptors. ACTB was included as a positive control.

Transcripts for *BMP2 *and *BMP4 *were readily detectable in elongated conceptuses and CT1 cells (Figure 1A). Transcripts for *NOG *were detected in elongated conceptuses but were either absent or detected at very low levels in CT1 cells (Figure 1A). Ectoderm and mesoderm expression of *NOG *is evident during gastrulation in other species [20], therefore it seems likely that the trophectoderm is not a primary source of *NOG*. It is interesting that transcripts for *NOG *could not be amplified from bovine endometrium (Figure 1A). Transcripts for *NOG *also could not be detected in other endometrial preparations obtained from cows in early pregnancy (days 14-17) (data not shown). Transcripts for *NOG *can be detected with in situ hybridization in mouse endometrium around the time of implantation, but the pattern of expression is restricted to stroma immediately underlying the epithelium [21]. Perhaps too little *NOG *mRNA exists in bovine endometrium for suitable amplification using conventional RT-PCR. Alternatively, perhaps the cow differs from mice and lacks endometrial NOG expression.

Transcripts for various BMP2 and 4 receptors subtypes also were detected in conceptuses and CT1 cells (Figure 1B). The TGFβ receptors are heterodimeric complexes comprised of two serine/threonine kinase receptor classes (type I and II). After ligand binding the type II receptor phosphorylates the type I receptor, which then initiates Smads and other intracellular transduction systems [22,23]. Bone morphogenetic proteins 2 and 4 interact with two specific type I receptors, termed BMPR1A (ALK3) and BMPR1B (ALK6), and a single type II receptor termed BMPRII [24,25]. Bone morphogenetic protein 2 also reacts with a third type I receptor subunit termed ACVR1 (ALK2) [26]. Transcripts for each of these receptor subtypes were detected in elongated conceptuses and endometrium (Figure 1B). The CT1 cells also contained the receptor machinery needed to respond to BMP2 and 4, although they appeared to contain very little *BMPR1B*. This receptor subtype is not required for normal placental formation in mice [27].

**Figure 2:**
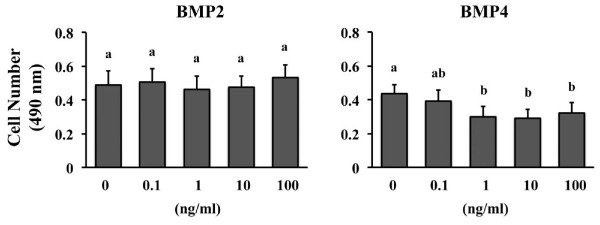
**Effect of BMP2 or BMP4 supplementation on CT1 cell survival**. CT1 cells were supplemented with various concentrations of BMP2 (left panel) or BMP4 (right panel) for 48 h, then viable cell numbers were quantified by examining the metabolism a tetrazolium compound (MTS) into a colored formazon product visible at 490 nm (n = 4 replicate studies for each BMP). Different superscripts within each BMP treatment denote differences (P < 0.05).

New roles for BMP4 in trophoblast lineage specification have emerged in the past several years. Trophoblast lineages are generated from human ESCs by BMP4 supplementation [28,29] and trophoblast development from bovine blastocyst outgrowths is stimulated by BMP4 [11]. However, potential functions for BMP2 and 4 after trophoblast specification have not been examined in cattle. The first study in this series of experiments examined whether BMP2 or BMP4 supplementation influenced CT1 cell growth (Figure 2). Supplementation with BMP2 did not affect viable cell numbers, but supplementation with 1, 10 or 100 ng/ml BMP4 reduced (P < 0.05) CT1 cell number after 48 h (Figure 2). Cells treated with BMP4 did not display any overt microscopic evidence of apoptosis or necrosis (detached, swollen/shriveled or punctated cells). Follow-up studies to quantify apoptosis and proliferation rates were not completed.

Another set of studies examined whether CT1 cells underwent any developmental modifications after supplementing serum free medium with 0.1, 1, 10 or 100 ng/ml BMP2 or BMP4. Microscopic examination of CT1 cells in various studies failed to detect changes in the incidence of BNC formation (< 2% in all groups; data not shown). Moreover, *CSH1 *transcripts could not be detected in CT1 cells after 48 or 96 h supplementation with BMP2 or 4 (data not shown). A mid-gestation placental RNA preparation was used to verify that CSH1 primers worked properly. The lack of detectable changes in trophoblast morphology and differentiation-dependent gene expression changes suggests that these factors may not be directly linked with trophoblast differentiation. However, limited effort has been devoted to uncovering ways to maximize BNC formation and BNC-specific gene expression in these cells. Therefore, a possible association between these BMPs and bovine trophoblast differentiation cannot be dismissed.

**Figure 3:**
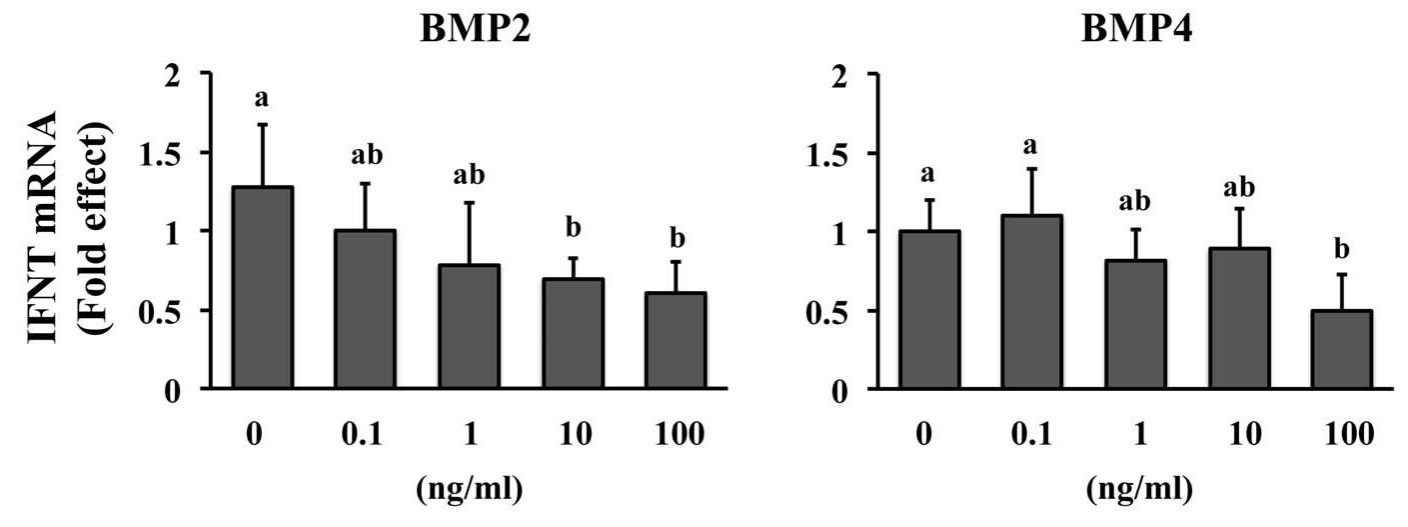
**Effect of BMP2 and BMP4 supplementation on IFNT mRNA abundance in CT1 cells**. Cells were supplemented with various concentrations of BMP2 (left panel) or BMP4 (right panel) for 24 h, then RNA was extracted and qRT-PCR was completed. 18 s RNA was used as the reference control. Data are presented as fold-differences from non-treated controls (n = 4 replicate studies for each BMP). Different superscripts within each BMP treatment denote differences (P < 0.05).

A final study determined that exposure to BMP2 or 4 decreased (P < 0.05) the relative abundance of *IFNT *mRNA (Figure 3). The minimal effective concentration needed to achieve this effect was less for BMP2 (10 ng/ml) than BMP4 (100 ng/ml). Expression of *IFNT *was decreased in a dose-dependent manner by BMP2 but not by BMP4. These outcomes suggest that BMP2 may be a more potent inhibitor of *IFNT *expression than BMP4. Changes in the synthesis and secretion of IFNT protein were not determined since *IFNT *mRNA abundance is usually reflective of protein production [16]. Interferon-tau exhibits a biphasic expression pattern during early pregnancy, where the amounts of IFNT mRNA increase dramatically around the time of conceptus elongation (day 13-15 in cattle) and decrease rapidly approximately one week later as implantation begins [30]. Several uterine- and conceptus-derived factors such as fibroblast growth factors (e.g. FGF2, 10) and colony stimulating factor 2 stimulate IFNT production from bovine trophectoderm [16,18,31,32]. These and other factors may play a part in the rapid increase in IFNT production during elongation. The identity of uterine and conceptus factors functioning as negative regulators of IFNT expression remained undetermined. Perhaps BMP2 and 4 may function as implantation-dependent down regulators of IFNT expression. However, the magnitude of the reduction in IFNT mRNA was not great (< 2-fold reduction) and it is not clear whether the large amounts of BMP supplementation needed to detect this effect have any physiological relevance.

In conclusion, ligands and receptors for BMP2 and 4 are expressed in elongating conceptuses and the CT1 cell line. No detectable changes in cell morphology or differentiation were detected after CT1 exposure to these BMPs. However, supplementation with BMP2 or 4 decreased cell growth rates and *IFNT *mRNA abundance. These observations implicate BMP2 and 4 as uterine and conceptus mediators of trophoblast development and IFNT production around the time of implantation.

## Competing interests

The authors declare that they have no competing interests.

## Authors' contributions

KAP assisted in the design of the studies, completed each of the studies, participated in the analysis and interpretation of the findings, and drafted the manuscript. ADE acquired funding for the project, assisted in the design of the studies, participated in the analysis and interpretation of the findings and generated the final draft of the manuscript. All authors read and approved the final manuscript.

## Author information

Current address for Dr. Kathleen Pennington; Division of Reproductive and Perinatal Research, Department of Ob-GYN and Women's Health, University of Missouri, Columbia, MO 65212
